# Comparative Analysis of Thermal Comfort and Antimicrobial Properties of Base Fabrics for Smart Socks as Personal Protective Equipment (PPE)

**DOI:** 10.3390/ma18030572

**Published:** 2025-01-27

**Authors:** Farhana Momotaz, Rachel Eike, Rui Li, Guowen Song

**Affiliations:** 1 School of Human Science, Mississippi State University, Starkville, MS 39762, USA; 2 Department of Apparel, Events, and Hospitality Management, Iowa State University, Ames, IA 50011, USA; rjeike@iastate.edu (R.E.); ruili@iastate.edu (R.L.); gwsong@iastate.edu (G.S.)

**Keywords:** personal protective equipment, thermal comfort, antimicrobial activity, smart socks, air permeability, moisture management

## Abstract

This study investigates the unique interplay between thermal comfort and antimicrobial properties in base fabrics, shaping the foundation for the development of “Smart Socks” as advanced personal protective equipment (PPE). By delving into the inherent qualities of fibers such as cotton, polyester, bamboo, and wool and exploring fabric structures like single jersey, terry, rib, and mesh, the research captures the dynamic relationship between material composition and performance. Terry fabrics emerge as insulators, wrapping the user in warmth ideal for cold climates, while mesh structures breathe effortlessly, enhancing air circulation and moisture wicking for hot environments. Cotton mesh, with its natural affinity for moisture, showcases exceptional moisture management. Antimicrobial testing, focused on fabrics’ interactions with Staphylococcus aureus, highlights the dormant potential of bamboo’s bio-agents while revealing the necessity for advanced antimicrobial treatments. This study unveils a vision for combining innovative fabric structures and fibers to craft smart socks that balance thermal comfort, hygiene, and functionality. Future directions emphasize sensor integration for real-time physiological monitoring, opening pathways to revolutionary wearable PPE.

## 1. Introduction

Footwear, particularly socks, is fundamental for maintaining foot health, which is essential for overall body alignment, posture, and musculoskeletal well-being. Foot health influences broader physical well-being, with improper footwear linked to conditions such as lower back pain, varicose veins, and even cardiovascular issues [1,2]. Socks, as a foundational element of footwear, significantly impact thermal regulation and moisture management, which are critical for foot health, particularly during prolonged standing or physical activity [3,4,5]. In occupational settings (e.g., firefighting, battlefield combating) and extreme environments, socks serve as a critical component of personal protective equipment (PPE) to maintain wearers’ safety and health [6,7].

Socks are clothing items primarily worn inside shoes and as such, they restrict ventilation more than garments used on other parts of the body, such as gloves, shirts, pants, and skirts [6]. They play a crucial role in foot health and comfort by influencing thermal balance and moisture transfer, acting as both external clothing and a barrier against cold conditions [6,7]. Thermal comfort in textiles is influenced by air permeability, thermal resistance, moisture management, water vapor resistance, fabric thickness, etc. [8]. These parameters collectively influence global comfort by balancing heat retention, breathability, and moisture control. The interplay between fabric structure (e.g., terry, mesh) and fiber type (e.g., cotton, wool, polyester) further determines how each parameter contributes to thermal and overall comfort. These attributes are especially critical for wearable garments like socks used in varying environmental conditions. If the fiber content and fabric structure of socks are not optimized, they may hinder the body’s ability to regulate temperature under varying environmental conditions, negatively impacting vital bodily functions [6].

Human bodies generate heat during activities such as exercising, walking, or even resting, with the rate of heat production aligning with perspiration [6]. Perspiration acts as a cooling mechanism, allowing water vapor to carry away surplus heat from the skin or fabric surface. Blood vessels in the ankles and feet play a significant role in regulating tissue temperature by constricting circulation during cold stress or increasing blood flow during warm conditions. However, excessive blood flow can lead to undesirable effects such as swollen feet [9]. These findings highlight the critical role of socks’ fiber content and fabric structure in maintaining health and thermal comfort.

The advancement of wearable technologies, particularly in healthcare and PPE, has opened opportunities for sensor integration into clothing. Smart PPE refers to wearable garments embedded with sensors for monitoring physiological parameters like temperature, pressure, or moisture. These innovations enhance the functions and performance of PPE for occupational and health applications [10]. Smart socks equipped with sensors can revolutionize foot health management by monitoring parameters like pressure and temperature, aiding in preventive and corrective care [11,12]. However, the performance of these wearables is often limited by their textile substrates, which must balance thermal comfort, moisture management, and durability [6,9].

Despite their potential, current smart garments typically lack fully embedded sensor technology, leading to discomfort, structural instability, and limited durability due to external attachments. Additionally, issues like poor washability and performance degradation under environmental stress hinder broader adoption [10,13,14]. Research into fibers and textile structures has demonstrated their significant impact on thermal management properties such as thermal resistance, water vapor permeability, and overall moisture management capacity [6,8]. For instance, polyester fabrics exhibit better moisture management due to their hydrophobic properties, while bamboo–cotton blends show high water vapor conductivity [15].

Studies also highlight the influence of fiber and fabric structure on thermal and antimicrobial performance. Terry fabrics are known for their superior thermal resistance and water vapor resistance, making them suitable for cold conditions, while mesh fabrics excel in air permeability and moisture management, ideal for hot environments [16].

Additionally, bamboo fibers exhibit natural antimicrobial properties due to “Bamboo Kun”, which inhibits the growth of microorganisms like *Escherichia coli* and *Staphylococcus aureus* [17].

The heels, soles, and toes of socks endure the highest abrasion and pressure. Research recommends using Lyocell or bamboo fibers in these areas for their antimicrobial activity and resistance to wear [17]. However, gaps remain in understanding the integration of sensors into socks to optimize comfort, durability, and health monitoring performance.

This study seeks to address these gaps by focusing on the foundational textiles for smart socks. Given the critical role of socks in health and thermal comfort, this study selected eight fiber types and four fabric structures to explore their influence on the thermal management and antimicrobial properties of socks intended for PPE applications. Antimicrobial activity was also evaluated due to its relevance for socks exposed to human perspiration, which is especially critical for PPE users. It evaluates a range of fibers and fabric structures for thermal resistance, moisture management, air permeability, and antimicrobial properties. By emphasizing the interplay between material composition and performance, this research identifies optimal configurations for sensor integration, paving the way for next-generation wearable PPE.

By bridging textile science with thermal comfort management, this study contributes to the innovative design of socks that prioritize user comfort, durability, and health monitoring capabilities. These findings advance the development of smart garments for occupational and medical applications, aligning with broader goals of enhancing individual well-being through the thermal management of substrates used for wearable technology.

## 2. Materials and Methods

In this study, a factorial experimental design is proposed to examine heat and moisture balance with the effects of fiber and fabric structure, as guided by methods and findings from studies conducted by Van Amber et al. (2015) [16] and Dhanapal and Dhanakodi (2020) [6] while also including antimicrobial qualities as informed by Arafa Badr (2018) [17].

### 2.1. Materials

The fiber content (i.e., base sock fabric component) of textiles proposed for exploration in this study include wool [16], polyester [6], bamboo [15,17,18], cotton [6,15], SeaCell/S–Lyocell [15], micro–modal/modal–rayon (M–rayon) [18], Tencel/Lyocell [17], and Lycra/elastane [17]. The details list is in Table 1. However, the list was adjusted based on the market availability of all the fiber content options listed with the target fabric structure (i.e., knitted configuration options). Specifically, wool and bamboo knit mesh fabrics were unavailable, and only a single jersey was available for SeaCell/S–Lyocell fabric. S–Lyocell is a biodegradable fiber that combines Lyocell (similar to TENCEL™) with seaweed (kelp) as an additive. Hence, this study refers to this fiber as seaweed–Lyocell (S–Lyocell) fabric to differentiate it from Tencel/Lyocell. Although we tried to collect 100% single fiber composition with four different structures, because of market unavailability in specific structure compositions, 27 distinct varieties of PPE sock fabrics were collected from the market, each with its own set of fibers, composition, structure, and yarn type which has been presented in Table 1.

The properties of textiles for PPE sock development have been explored by testing the PPE’s moisture management and thermal properties to see which fiber composition, structure, and yarn types provide the best comfort when used as PPE. After the initial tests were performed, select textiles were then explored regarding their antibacterial characteristics. This phased approach to exploring moisture and thermal management properties first, then selecting only those with the highest performance ratings for antimicrobial testing, was simply due to efficiently managing resources. Lastly, based on the findings of fiber, structure, and composition discovered during this research, a digital prototype design of smart sock PPE is proposed with the aims of informing and assisting the next phases of this research agenda to develop “PPE Smart Socks” and provide better comfort, performance, and overall health for users.

### 2.2. Test Methods

To measure thermal and moisture management properties, such as absorption rate, maximum wetted radius, spreading speed, cumulative one-way transport index, overall moisture management capability, thermal conductivity, water vapor transmission, air permeability, and antimicrobial properties and to see which fiber composition and structure provide the best performance for PPE sock application, the test methods (TMs) described below are followed in this study (see Table 2). The specimens were tested under the standard atmosphere for testing at 21 °C and 65% humidity. The researchers conducted 3 replications of each textile sample for each test method for all 27 specimens from each type of fabric composition. After calculating the specimens, the mean and the standard deviation were calculated to determine a standard number.

The data collected from all the outlined standardized test methods were analyzed by performing univariate analysis of variance (ANOVA) routines using IBM SPSS Statistics 29.0.0.0 to identify significantly different values per variable in the experimental design. Also, based on the result of each test, a post hoc comparative analysis of the effect of different fiber compositions and fabric structures on thermal, moisture, and other functional characteristics was conducted. Bivariate correlation analysis helped find any significant correlation among the variables.

## 3. Results

In this section, the data gained from all the test results have been analyzed to determine the relationship of the variables and demonstrate the factors that affect the thermal and comfort properties of different fibers and structure composition.

Table 3 shows that most of the terry structures have the highest thickness among the eight fibers with four different fabric structures, followed by rib, single jersey, and mesh. However, the thickest fabric is wool rib (2.6 mm), followed by wool terry (1.42 mm), Lyocell terry (0.95 mm), cotton terry (0.84 mm), and elastane single jersey (0.816 mm). The thinnest fabric is Lyocell mesh (0.196 mm), followed by elastane mesh (0.2 mm).

Table 3 also shows that wool rib (697.7 g/m^2^) has the highest mass per unit weight, followed by Lyocell terry (352.3 g/m^2^), bamboo rib (327.3 g/m^2^), and wool terry (324.3 g/m^2^). On the other hand, Lyocell mesh (62.7 g/m^2^) has the lowest mass per unit weight, followed by elastane mesh (89.7 g/m^2^) and cotton mesh (104.7 g/m^2^). Therefore, it can be assumed that mesh fabric has the lowest thickness, whereas rib and terry have the highest thickness.

Air permeability is an important factor influencing thermal comfort, as it governs the rate at which air passes through the fabric, thereby affecting heat dissipation and insulation. High air permeability enhances ventilation, facilitating the removal of excess heat and moisture from the skin’s surface, which is critical for maintaining thermal comfort in warm environments. From the air permeability test, it has been shown in Table 4 that cotton mesh (622 cm^3^/cm^2^/s) has highest air permeability, followed by elastane single jersey (527 cm^3^/cm^2^/s), Lyocell mesh (471 cm^3^/cm^2^/s), polyester rib (229 cm^3^/cm^2^/s), wool terry (223 cm^3^/cm^2^/s) and Lyocell single jersey (217 cm^3^/cm^2^/s). Cotton terry has the lowest air permeability (15 cm^3^/cm^2^/s), followed by bamboo terry (46 cm^3^/cm^2^/s) and Lyocell terry (67 cm^3^/cm^2^/s). This result is due to the inherently open structure of mesh fabrics, allowing for unobstructed airflow. In contrast, the dense and looped terry structures create multiple barriers to airflow, compounded by their increased thickness. These structural differences align with the requirements for different environmental conditions, where mesh provides ventilation in hot climates and terry offers insulation in colder settings.

Previous studies showed that fabric thickness significantly affected the socks’ air permeability values, since air permeability tended to decrease as thickness increased, irrespective of fiber type [18]. However, the results of this study, after performing a bivariate correlation analysis between thickness and air permeability, we have found that the R^2^ value for polyester is 0.16, cotton 0.08, wool 0.08, elastane 0.88, Lyocell 0.75, M–rayon 0.65, and bamboo 0.54. S–Lyocell only has one structure, so it was not possible to find any correlation between thickness and air permeability. Therefore, it can be suggested that there is a strong correlation between the thickness and air permeability of elastane, Lyocell, M–rayon, and bamboo fabric.

**Figure 1:**
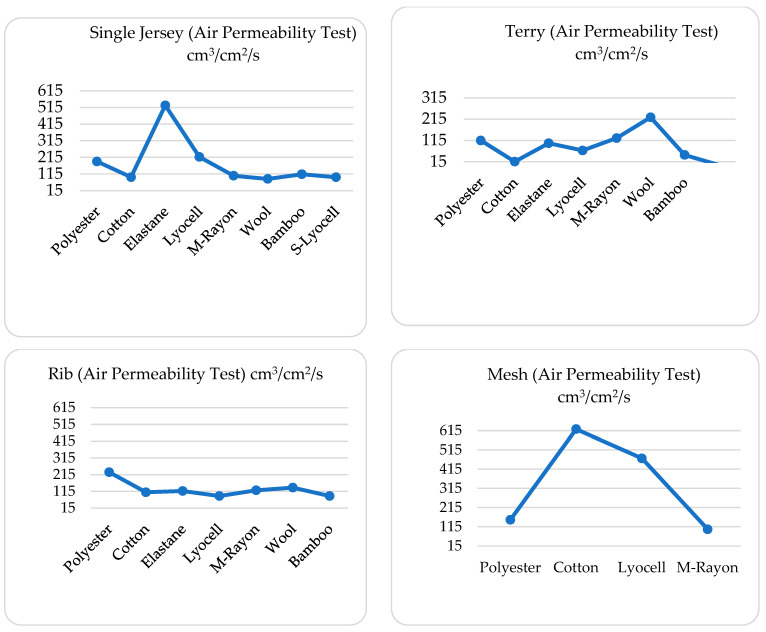
Air permeability of different fabric structures.

From the above graph in Figure 1, data indicate that air permeability is consistent with that of four different structures and eight fiber types. The acceptable range of air permeability is defined as 40 to 200 cm^3^/cm^2^/s [26], where a majority of samples from this project fell within this range. From the test, elastane single jersey (527.5) has a very high AP value, which is beyond the range, probably because of its increased loop length. This textile structure is followed by Lyocell, polyester, and bamboo, which are in the acceptable range. Researchers have found that increased loop length causes higher AP [27]. From the terry structure, cotton terry has very low air permeability (15) and wool has high AP (223, i.e., slightly higher than the range). This is likely due to the cotton fiber characteristics (convolutions in the structure with the striations over the longitudinal surface) that led to these results. A previous study suggested that the increased friction between the fiber surface and the air results in a decrease in the air permeability of fabrics from these fibers [18], which further supports the results of this research. M–rayon, polyester, elastane, and bamboo then followed the air permeability ranking of cotton terry and wool. Polyester has a high AP (slightly more than good AP value) in the rib structure, followed by wool, M–rayon, and elastane. Cotton has the highest AP (622) in the mesh structure, followed by Lyocell (441), which is so high and beyond the range probably because of its porous structure. Polyester and M–rayon mesh structures have acceptable AP values. Elastane gives no results, probably because of its low porosity and thickness. It has been found in previous research that low porosity may affect the air permeability value, which further supports this result [28,29].

Although some studies identify fabric thickness as a factor affecting some comfort and moisture properties of sock fabrics [18], this study found no significant correlation between the selected textiles’ thermal properties and thickness. However, this study found some significant correlation between the fabric structure and thermal properties, particularly air permeability. From this study, it was found that there is a strong correlation between thickness and air permeability when looking at the textiles researched. If thickness increases, air permeability decreases. Because of higher thickness, most terry structure fabrics have lower air permeability, while most mesh structures have higher air permeability.

The moisture management test results in Table 5 show that cotton mesh has the best overall moisture management capacity (OMMC) value, i.e., 0.83 (Grade 5) because of its fiber content and low thickness, whereas wool rib has the worst OMMC value, i.e., 0 (Grade 0) because it has the highest thickness that the moisture could not transfer to the other side of the fabric. Cotton terry, M–rayon single jersey, and wool single jersey have a bad OMMC value (Grade 1). Polyester terry, polyester rib, cotton single jersey, elastane mesh, Lyocell terry, Lyocell mesh, M–rayon terry, and bamboo terry have a good OMMC value because of their low thickness and higher moisture transfer rate (Grade 4).

One way-transport index grade values are high for polyester rib (1170), polyester terry (1154), cotton mesh (1008), and M–rayon terry (1007), which means that these fabrics can transmit sweat to the outer side much faster than others, which is a very important property for socks that aim to be used for personal protective or preventative purposes.

From this study, after performing bivariate correlation analysis between fabric thickness and OMMC, it was found that the R^2^ value for polyester is 0.29, cotton 0.56, elastane 0.52, Lyocell 0.0041, M–rayon 0.01, wool 0.35, and bamboo 0.11. The overall correlation is 0.25, which is very low. Therefore, it can be suggested that the OMMC properties of the fabric samples have very little correlation with their thickness.

The overall moisture management capacity (OMMC) is a composite metric that quantifies the dynamic interaction between moisture absorption, spreading, and evaporation within a fabric. This parameter directly relates to the comfort sensation by assessing the ability of a textile to manage perspiration effectively. Higher OMMC values indicate superior performance in transporting sweat away from the skin to the outer fabric surface, where it can evaporate, thus ensuring a dry and comfortable microenvironment. In the study, fabrics like cotton mesh, with its porous structure and hydrophilic properties, demonstrated the highest OMMC, enhancing moisture wicking and evaporation. Conversely, dense structures like wool terry exhibited lower OMMC values, limiting moisture movement and potentially causing discomfort in humid conditions. These findings underscore the critical role of fabric design in optimizing moisture management and comfort in wearable PPE applications.

Table 6 shows that the average thermal resistance (RCF) value of wool terry, 0.13, is the highest, followed by cotton terry (0.04), wool single jersey (0.03), and Lyocell terry (0.03). The lowest average RCF value is elastane mesh (0.004). The higher the thermal resistance, the better the insulation in a cold environment. This is attributed to the thicker and denser looped structure of terry fabrics, which traps air within the fabric matrix. Air is a poor conductor of heat, enhancing the insulating properties of these fabrics. Wool, a natural protein fiber, further contributes to thermal resistance due to its crimped structure, which increases bulk and air retention combined with its low thermal conductivity. Therefore, it can be determined that wool terry can trap more heat, followed by cotton terry, wool single jersey, and Lyocell terry, whereas the elastane mesh can trap less heat. Therefore, in colder environmental weather conditions, the skin’s surface may get cold more quickly, and wool terry may be a good fiber and structure to entrap heat produced by the body to keep it insulated for warmth. Conversely, elastane mesh has shown to be a strong conductor of heat, as it draws heat away from the skin to keep the body cool, making this fiber and structure a more comfortable choice for use in a warm-to-hot environment.

Table 6 also represents the water vapor resistance test value (REF). It has been found that the highest average REF value is for wool terry (11.7), followed by cotton terry, bamboo rib, and Lyocell terry. The lowest REF is Lyocell rib (0.5), followed by cotton rib (0.7) and polyester single jersey (0.7). The wool rib had an error in the result because the fabric was so thick that it could not meet the minimum requirement in the machine. REF determines a fabric’s ability to allow water vapor (perspiration) to pass through it. The lower the value obtained, the more breathable the fabric. Therefore, it can be determined that Lyocell rib is the more breathable fabric, followed by cotton rib, polyester single jersey, polyester rib, and polyester mesh.

Equation of total thermal loss:

$$Total\;Thermal\;loss:Qt=\frac{10\;{}^{\circ}C}{\left(RCF+0.04\right)}+\frac{3.57\;KPa}{\left(REF+0.0035\right)}$$

where

Qt = total heat loss (W/m^2^);
RCF = average intrinsic thermal resistance of the laboratory sample (K.m^2^/W);
REF = average apparent intrinsic evaporative resistance of the laboratory sample (Kpa.m^2^/W).

Total thermal loss refers to the decrease in heat existing in space in both dry heat loss means such as convective and radiative heat transfer and wet heat loss means such as evaporative heat loss. In clothing, thermal loss can be described as the process of heat transferring from the body through single or multiple layers of clothing to the outside environment. Heat loss occurs when there is a temperature difference between two environments (near body and external to body), and heat flows spontaneously from the warmer environment to the colder environment. The amount of heat loss depends on the surface area through which the heat flows, the material/textile, and the temperature difference. Thermal resistance (R) is the ability of an object to impede heat transfer by way of conduction through a given thickness of the substance. Mathematically, thermal resistance (R) = L/K; L = insulation thickness, K = thermal conductivity. So, if the thermal loss is low, thermal resistance could be high and the conductivity will be low [30].

From Table 7, Lyocell rib has the highest thermal loss (1054), meaning more conductivity and the lowest thermal resistance, followed by polyester S/J (1050) and polyester mesh (995), whereas wool terry (294.3) has the lowest thermal loss as well as low conductivity and highest thermal resistance, followed by cotton terry (557) and Lyocell terry (617).

**Figure 2:**
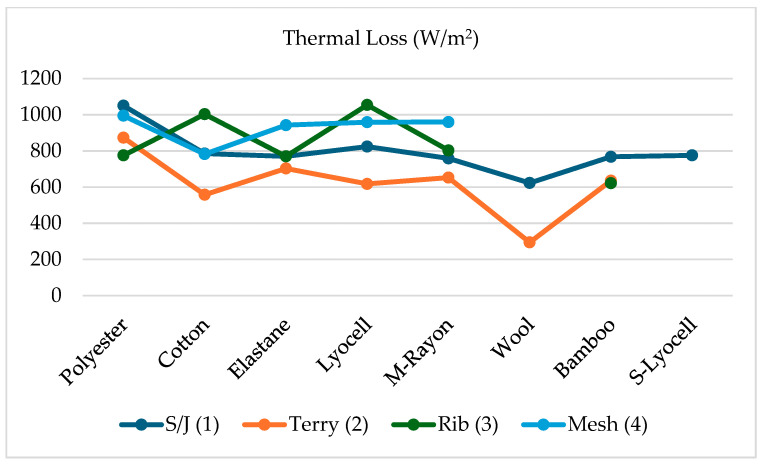
Thermal loss of different fabric structure and fiber content.

According to previous research, terry fabrics were the most thermal and water vapor resistant, least permeable to water vapor, most absorbent, and most conductive [18]. They were also the thickest and had the highest mass of all the fabrics. Single jersey fabrics were the thinnest, lightest, and were the least thermal and water vapor resistant, most permeable to water vapor, least absorbent, and least conductive. It is generally acknowledged that thicker fabrics are most resistant to both heat and water vapor transfer [18]. In this study, from Figure 2, terry fabrics show the lowest thermal loss, whereas most of the mesh fabrics have higher thermal loss. That means terry fabric has low conductivity and high thermal resistance, as well as high water vapor resistance compared to single jersey, rib, and mesh because of their thickness. In contrast, most of the mesh fabrics have high thermal and water conductivity because of high thermal loss and low resistance, probably because of their porous structure.

Table 8 shows that wool single jersey has the highest amount of water vapor transmission (35.02), followed by elastane single jersey (32.52) and cotton terry (31.98), while modal–rayon rib has the lowest amount of water vapor transmission (20.74).

**Figure 3:**
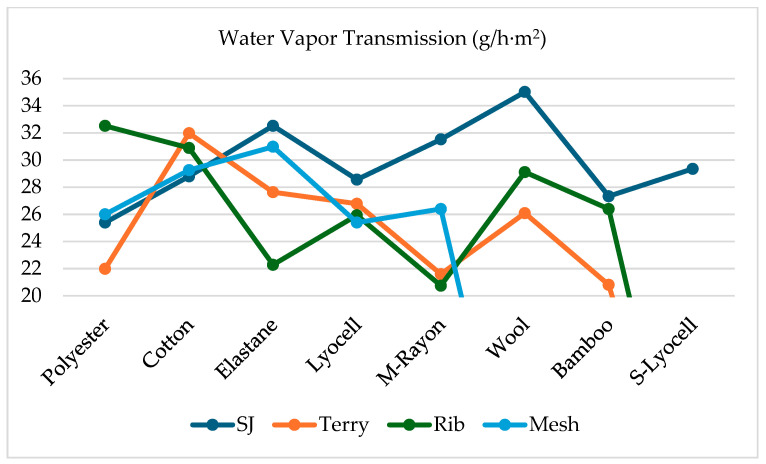
Water vapor permeability/transmission.

In previous research, water vapor transmission (WVT) has been reported as lowest in bulky and hairy fabrics and highest for fabrics with open structures (e.g., large inter-yarn and interstitial spaces) [16]. In this study, terry fabrics show the lowest WVT (Figure 3) probably because of their thickness and structure, which can trap a greater volume of fibers and air through which the water vapor must permeate, thus creating a stronger resistance to heat and water vapor. However, its construction is more open than single jersey. Although some studies identified fabric thickness as a factor affecting some comfort and moisture properties of sock fabrics [18], this study found no significant correlation between the thermal properties of socks and thickness. Still, there is some significant correlation between the fabric structure and thermal properties.

In summary, regarding fibers, polyester, cotton, wool, and bamboo, these have acceptable and higher air permeability. Therefore, this could be a consideration for PPE sock fabrication, since higher air permeability helps to make it a breathable fabric. From this study’s moisture management test results, it can be determined that cotton mesh has the highest OMMC value, while wool terry showed the lowest. The superior OMMC of cotton mesh is due to the open and porous structure of the mesh fabric, which facilitates rapid moisture transport and evaporation. Cotton fibers, being hydrophilic, enhance the absorption of sweat. In contrast, wool terry has a denser structure and hydrophobic surface scales that hinder the rapid movement of moisture, leading to lower OMMC values. Polyester terry, polyester rib, cotton single jersey, and bamboo terry have good OMMC values. Polyester rib, polyester terry, and cotton mesh have higher one-way-transport index grade values, which means that these fabrics can transmit sweat to the outer side much faster than others, which is a very important property for PPE socks. From this study, the water resistance test represents the highest average REF value for wool terry, followed by cotton terry and bamboo rib. Therefore, they could be a good option for PPE garments in extremely hot and humid weather.

As described previously, select textiles with the highest performance ratings were chosen based on their overall moisture management, air permeability, and breathability from the thermal comfort phase of the study to advance onto the antimicrobial testing simply due to efficiently managing resources. Therefore, cotton, wool, bamboo, and polyester fiber were selected to go through the antimicrobial test following the AATCC Test Method 147 using *Staphylococcus aureus* (ATCC 6538) as the microbial agent on nutrient agar with samples of the select fabrics. Only rib structures of the above four fiber contents were tested for antimicrobial tests because of their possibility to show higher antimicrobial properties than other structures [17].

**Figure 4:**
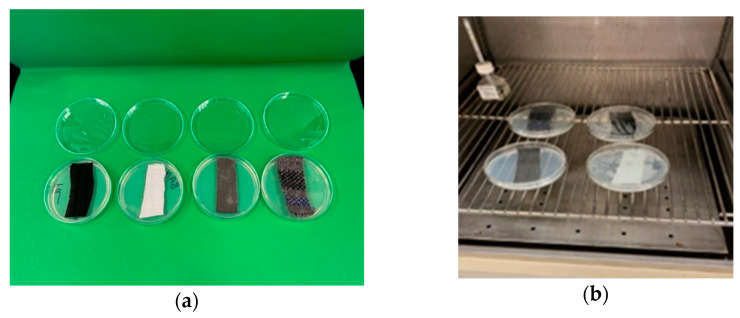
Antimicrobial test samples of fabrics on inoculated nutrient agar plates before (**a**) and during (**b**) incubation at 37 °C.

After incubation for 24 h, the *S. aureus* grew and formed colonies along the streaked areas on the agar surface. The density of colonies decreased progressively from the first to the fifth streak. This was due to fewer cells of the organisms deposited on the agar with subsequent streaks. (Note: this is a dilution effect.) Generally, no growth inhibition (clear zones) of *S. aureus* along the streaks near the fabrics was observed (Figure 4a).

One likely reason for this lack of inhibition is that the antimicrobial agent (if present) was not diffusible in agar and remained bound to the fabric. However, viewing the agar plates’ underside, it was observed that *S. aureus* grew in the agar directly in contact with the fabrics. Moreover, bacterial contaminants (larger colonies) of the fabrics also grew on the agar underneath each fabric (Figure 4b).

**Figure 5:**
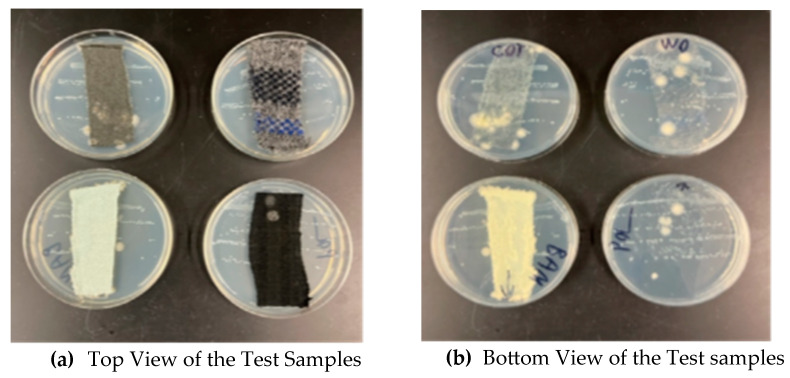
Growth of *Staphylococcus aureus* (ATCC 6538) on nutrient agar with samples of fabrics.

Figure 5a,b represents the top view (a) and bottom view (b) of the agar plates with the fabrics. From left to right, fabrics in the top row are cotton and wool; those in the bottom row are bamboo and polyester. Colonies of *S. aureus* can be seen along the streaks on the agar surface. Large cream-colored colonies are from microbial contaminants on the fabrics.

Based on the AATCC Test Method 147, no antimicrobial activity of the fabrics was observed against *S. aureus* (ATCC 6538). If the fabrics have antimicrobial properties, it is likely that the concentration of the antimicrobial agent is lower than the minimum inhibitory concentration for the *S. aureus* used in the present study. However, a previous study by Arafa Badr (2018) explored that bamboo samples had the strongest antimicrobial activity against *Escherichia coli* (G−) and *Staphylococcus aureus* (G+) because of the presence of a bio-agent called “Bamboo Kun” in the bamboo fiber that provides natural antimicrobial activity in its structure to prevents microorganisms from growing and repels the colonization of *E. coli* and *S. aureus* [17]. However, this study contrasts with the previous study’s findings, probably because the “Bamboo Kun” was not diffused with the solution and bound strongly with the fabric, hindering the antimicrobial effect.

To explore the correlation between thermal comfort and antimicrobial properties, bivariate correlation analysis was conducted. The results indicate no significant direct correlation between the two properties across the fabric types and structures tested. This lack of correlation is attributed to the independent mechanisms governing these properties. Thermal comfort is largely influenced by fabric thickness, structure, and air permeability, which affect heat and moisture transfer. Conversely, antimicrobial properties depend on factors such as fiber composition and the presence of antimicrobial agents.

For instance, while bamboo fiber demonstrated potential antimicrobial properties due to its natural bio-agent “Bamboo Kun”, it did not exhibit superior thermal comfort metrics compared to other fibers like polyester or wool. Similarly, terry structures, known for their excellent thermal resistance, did not inherently display antimicrobial activity unless treated with external agents. These low correlation values highlight the distinct nature of the factors influencing thermal comfort and antimicrobial performance, underscoring the need for multi-functional material development for PPE applications.

There appears to be no significant direct correlation between moisture management and antimicrobial properties. The two properties are governed by independent mechanisms: moisture management is influenced by fabric structure, fiber composition, and thickness, while antimicrobial properties depend on factors such as the presence of antimicrobial agents and the fiber’s natural characteristics.

From the antimicrobial tests performed using *Staphylococcus aureus* (ATCC 6538), fabrics such as bamboo exhibited potential due to the natural bio-agent “Bamboo Kun”, but this did not directly translate to superior moisture management. For example, cotton mesh, which had the highest overall moisture management capacity (OMMC) of 0.83 (Grade 5), did not show significant antimicrobial activity. Conversely, rib structures with compact designs, selected for antimicrobial testing, displayed no clear zones of inhibition in the agar plate method, even when certain fibers had good moisture transport properties.

The lack of correlation is supported by the independent mechanisms influencing these properties. Statistical analysis also indicates no significant bivariate correlation between moisture management parameters (e.g., OMMC) and antimicrobial efficacy across fabric types and structures.

## 4. Discussion

The thermal resistance of fabrics plays a crucial role in maintaining user comfort by reducing heat loss, especially in cold environments. Previous research has highlighted the importance of balancing these properties to enhance overall thermal comfort in wearable textiles [31]. Therefore, considering this critical aspect of balancing user comfort and functionality, the overall findings of this study have outlined rankings of the top-performing textiles for each test method considering the thermal comfort of PPE socks (see Table 9). Organizing the findings this way allows for a quick review of the top textiles for suggested placements into knit-based smart clothing.

Polyester single jersey, polyester mesh, and wool rib have acceptable and higher air permeability. Therefore, this could be a consideration for PPE sock fabrication, since higher air permeability helps to make it a breathable fabric.

From the test result of this study, it can be determined that the overall moisture management properties of the fabric samples have very little correlation with their thickness. Cotton mesh has the highest OMMC value, but it is beyond the acceptable range. Polyester terry, polyester rib, cotton single jersey, elastane mesh, Lyocell terry, Lyocell mesh, M–rayon terry, and bamboo terry have good OMMC values. Polyester rib, polyester terry, cotton mesh, and modal–rayon terry have higher one way-transport index grade values, which means that these fabrics can transmit sweat to the outer side much faster than others, which is a very important property for PPE socks.

In this study, terry fabric has the lowest thermal loss, whereas most of the mesh fabrics have higher thermal loss. That means terry fabric has low conductivity, high thermal resistance, and high water vapor resistance compared to single jersey, rib, and mesh. The water resistance test represents the highest average REF value for wool terry, followed by cotton terry, bamboo rib, and Lyocell terry. Therefore, it can be suggested that terry fabrics could be used where a higher water resistance value is required. Water vapor resistance determines a fabric’s ability to allow water vapor (perspiration) to pass through it. The lower the value obtained, the more breathable the fabric. In this study, it has been found that terry fabric probably has the lowest WVT. Therefore, it could be an option when worn PPE products need stronger resistance to heat and water vapor to resist the outside temperature and liquid transmitted through the garments. However, Lyocell rib, cotton rib, polyester rib, and polyester single jersey are the more breathable fabrics because they have the lowest REF. Therefore, they could be a good option for PPE garments in extremely hot and humid weather.

Since most of the fibers did not show any antimicrobial activity, it can be suggested that until any antimicrobial finish is applied to the textiles, it does not represent any inhibition zone against human pathogenic bacteria *S. aureus*. However, based on previous studies by Arafa Badr (2018), it could be assumed that 100% bamboo fiber could exhibit some natural antimicrobial properties if no additional finishing is applied on the surface. The rib structure can potentially create more inhibition zones than the other three structures because of its compact structure [17].

The integration of sensors for physiological data collection into PPE socks is pivotal for enhancing their functionality beyond basic protection and comfort. Sensors embedded in PPE socks can monitor critical parameters such as foot pressure, temperature, and moisture levels, providing real-time data to assess the wearer’s physiological and environmental conditions.

These sensors are strategically placed in areas of high pressure, such as the toes and heels, to optimize data accuracy without compromising comfort. This study suggests using breathable fabrics like polyester rib and single jersey or Lyocell rib at sensor integration points to ensure thermal and moisture management, preventing sensor degradation and maintaining wearer comfort.

**Figure 6:**
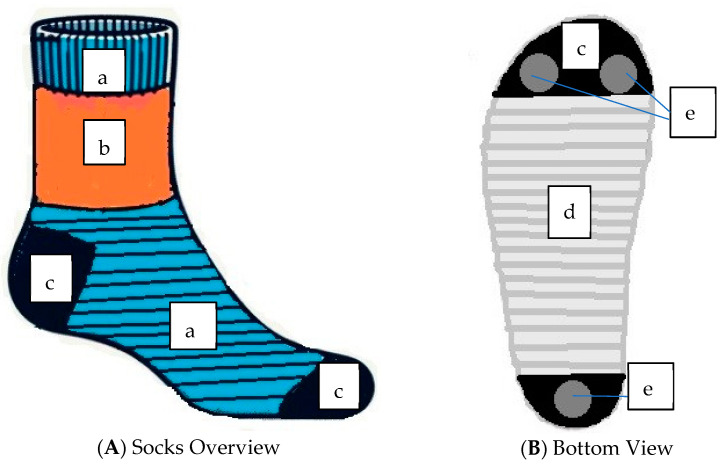
Technical drawing of smart sock PPE concept. (**A**) Sock overview; (**B**) bottom view. Notes: (a) polyester rib, (b) polyester SJ, (c) Lyocell rib, (d) bamboo rib, (e) sensors.

By enabling real-time monitoring, these smart PPE socks contribute to improved safety, health, and performance in occupational or medical settings, addressing issues like foot strain, overheating, or excessive moisture, which can lead to discomfort or injury. In relation to physiological data collection points, sensors sensitive to foot pressure could be placed on the toes and heel areas of the PPE socks because these areas mostly exert the highest pressure on the foot. Abrasion and pilling usually occur on the socks’ heel, sole, and toes. However, the sole part has the highest pilling and abrasion resistance grade, followed by the heel and toe [17]. Therefore, if sensors are placed in this area, especially the toe and heel, that would be helpful to collect physiological data (i.e., temperature and pressure) from the foot because of foot pressure. Since most polyester fabric structures, especially polyester rib and single jersey, have higher air permeability, very good OMMC, and good thermal and water resistance, they could be acceptable options for smart sock PPE. Polyester rib, Lyocell rib, and polyester single jersey are more breathable regarding thermal resistance, water vapor resistance, and water vapor transmission. Hence, they could be ideal fabric to place the sensor in the smart socks so that it is breathable and can transmit the excess moisture, vapor, and heat quickly so that the sensor does not get damaged because of the influence of the external environment. Figure 6 outlines the concept of how smart sock PPE could take shape.

Figure 6A shows the proposed smart sock PPE overview. Using polyester ribs (b) in the top and middle parts will contribute to the thermal management of the socks. Because of good air permeability, a polyester single jersey (a) could be used in the top part under the polyester rib edge. Lyocell rib (c) or the blend of polyester and Lyocell rib could be used in the toe and heel part, where pressure-sensitive sensors (temperature and pressure sensor) will be placed because of its good thermal loss, low water vapor resistance, breathability, and good strength. Two pressure-sensitive sensors (e) will be placed in the toe part and one sensor in the heel part of the bottom of the socks in Figure 6B because this part exerts the maximum pressure under the feet [32]. The sole part of Figure 6B could use 100% bamboo rib fiber to inhibit the growth of microbes. As this part of the foot usually does not go through huge abrasion, bamboo fiber will be a good fit for the sole part [17]. Bamboo ribs also have moderate thermal and water resistance and acceptable air permeability, making them a good fit considering their antimicrobial potential. These combinations could be a good fit for certain environmental conditions with high temperatures or average working conditions (~68–75 degrees F) or interior working environments for the occupational worker in a controlled/semi-controlled temperature or environmental space to provide maximum thermal comfort and breathability naturally.

It is important to restate that this figure is a conceptual image and that more research is needed to transition into prototype development. This study serves as the first phase of the larger PPE footwear research agenda.

The findings of this study are expected to advance the field of PPE and smart garment product development from the approach of textile details directly associated with footwear. As feet and ankles are highly complex in form and function and their ability to perform for work and enjoyment directly depends on high-quality footwear, including socks, we anticipate that this initial phase of research will inform and guide future phases that can have a positive meaningful impact on specialized groups of the medical, workforce, and social communities. The research team also anticipates that the findings of this study have the potential for product integration that involves other body areas/regions and into products for a variety of data collection purposes. One of the limitations of this study included the unavailability of certain fibers and textile compositions to conduct performance tests.

## 5. Conclusions

This study explored the thermal comfort, moisture management, and antimicrobial properties of various fibers and fabric structures to identify optimal substrates for the development of smart sock PPE. Findings indicate that fabric composition and structure significantly influence key performance metrics. Terry fabrics demonstrated high thermal resistance, making them suitable for cold environments, while mesh fabrics excelled in air permeability and moisture management, ideal for warm climates. Polyester fibers stood out for their superior moisture transport capabilities, and bamboo exhibited potential for antimicrobial activity, although further enhancement through finishing treatments is necessary.

The research underscores the importance of aligning textile properties with specific functional requirements for smart socks, including sensor integration. This foundational work provides critical insights into the interplay between material composition and wearability, laying the groundwork for next-generation PPE socks that balance comfort, durability, and health-monitoring capabilities. Future research should focus on integrating sensors into these optimized textile substrates to enable real-time physiological monitoring, further advancing wearable technology for occupational and medical applications.

## Figures and Tables

**Table 1:** Factorial design of the fiber and fabric combinations for the experiment.

Fibers	Tests and Structure of Fabric Samples							
	Air Permeability ASTM D737-18 [19]	Thickness ASTM D1777-96 [20]	Mass/Area BS EN 12127: 1998 [21]	Thermal Resistance ISO 11092: 1993(E) [22]	Water Vapor Resistance ISO 11092: 1993(E) [22]	Moisture Management AATCC 195 [23]	Water Vapor Transmission ASTM E96 [24]	Antimicrobial AATCC 147:2004 [25]
Cotton (Cellulose)	SJ, T, R, M	SJ, T, R, M	SJ, T, R, M	SJ, T, R, M	SJ, T, R, M	SJ, T, R, M	SJ, T, R, M	R
Bamboo (Cellulose)	SJ, T, R	SJ, T, R	SJ, T, R	SJ, T, R	SJ, T, R	SJ, T, R	SJ, T, R	R
Wool (Protein)	SJ, T, R	SJ, T, R	SJ, T, R	SJ, T, R	SJ, T, R	SJ, T, R	SJ, T, R	R
Polyester (Polyethylene terephthalate)	SJ, T, R, M	SJ, T, R, M	SJ, T, R, M	SJ, T, R, M	SJ, T, R, M	SJ, T, R, M	SJ, T, R, M	R
Elastane (Polyurethane)	SJ, T, R, M	SJ, T, R, M	SJ, T, R, M	SJ, T, R, M	SJ, T, R, M	SJ, T, R, M	SJ, T, R, M	
Modal–Rayon (Regenerated Cellulose)	SJ, T, R, M	SJ, T, R, M	SJ, T, R, M	SJ, T, R, M	SJ, T, R, M	SJ, T, R, M	SJ, T, R, M	
Lyocell (Regenerated Cellulose)	SJ, T, R, M	SJ, T, R, M	SJ, T, R, M	SJ, T, R, M	SJ, T, R, M	SJ, T, R, M	SJ, T, R, M	
Seaweed–Lyocell (Regenerated Cellulose)	SJ	SJ	SJ	SJ	SJ	SJ	SJ	

Note: fabrication knit structures are as follows: SJ = single jersey; T = terry; R = rib; and M = mesh.

**Table 2:** Details of test methods.

Performance Measure	Test Method	Test Specimen Count	Equipment
Thickness	ASTM D1777-96	27	Frazier Schiefer Compressometer, National Institute of Standards and Technology (NIST), Gaithersburg, MD, USA
Mass per unit area	BS EN 12127:1998	27	Precision balance or scale, Sartorius AG, Göttingen, Germany
Air permeability	ASTM D737-18	27	Air Permeability tester, SDL ATLAS M021A, Rock Hill, SC, USA
Moisture management test	AATCC 195	27	Moisture management tester, SDL ATLAS MMT M290, Rock Hill, SC, USA
Thermal resistance	ISO 11092:1993(E)	27	Sweating guarded hotplate, SDL Atlas, Rock Hill, SC, USA
Water vapor resistance	ISO 11092:1993(E)	27	Sweating guarded hotplate, SDL Atlas, Rock Hill, SC, USA
Water vapor Transmission	ASTM E96	27	Jar method, Thwing-Albert Instrument Company, West Berlin, NJ, USA
Antimicrobial activity	AATCC 147:2004	4	Parallel streak method from AATCC to evaluate the antimicrobial activity of selected fabrics against Staphylococcus Aureus, Fisher Scientific, Waltham, MA, USA

**Table 3:** Structural feature of the sock’s fabric.

Fiber and Structure	Single Jersey		Terry		Rib		Mesh	
	Thickness (mm)	Mass/Weight (g/m^2^)	Thickness (mm)	Mass/Weight (g/m^2^)	Thickness (mm)	Mass/Weight (g/m^2^)	Thickness (mm)	Mass/Weight (g/m^2^)
Polyester	0.51	200.00	0.48	208.00	0.43	176.00	0.73	239.00
	0.54	209.00	0.50	218.00	0.44	171.00	0.73	248.00
	0.52	199.00	0.48	206.00	0.43	172.00	0.73	249.00
AVG.	0.52	202.67	0.49	210.67	0.43	173.00	0.73	245.333
SD.	0.01	5.51	0.01	6.43	0.01	2.65	0.00	5.51
Cotton	0.43	164.00	0.60	290.00	0.46	194.00	0.30	102.00
	0.42	151.00	0.55	291.00	0.45	194.00	0.30	99.00
	0.42	154.00	0.54	298.00	0.44	193.00	0.29	113.00
AVG.	0.43	156.33	0.57	293.00	0.45	193.66	0.30	104.66
SD.	0.00	6.81	0.03	4.36	0.01	0.58	0.00	7.37
Elastane	0.82	216.00	0.60	248.00	0.46	194.00	0.30	86.00
	0.81	224.00	0.55	245.00	0.45	191.00	0.30	90.00
	0.83	200.00	0.54	229.00	0.44	190.00	0.29	93.00
AVG.	0.82	213.33	0.57	240.67	0.45	191.67	0.30	89.67
SD.	0.01	12.22	0.03	10.21	0.01	2.08	0.00	3.51
Lyocell	0.46	184.00	0.95	358.00	0.53	239.00	0.19	65.00
	0.45	186.00	0.94	351.00	0.57	247.00	0.21	61.00
	0.44	185.00	0.96	348.00	0.58	250.00	0.19	62.00
AVG.	0.45	185.00	0.95	352.33	0.56	245.33	0.20	62.67
SD.	0.01	1.00	0.01	5.13	0.02	5.69	0.01	2.08
M–Rayon	0.46	173.00	0.48	218.00	0.54	219.00	0.40	154.00
	0.46	183.00	0.50	229.00	0.60	219.00	0.41	155.00
	0.47	190.00	0.52	234.00	0.56	225.00	0.40	152.00
AVG.	0.46	182.00	0.50	227.00	0.57	221.00	0.40	153.67
SD.	0.01	8.54	0.02	8.19	0.03	3.46	0.00	1.53
Wool	0.53	226.00	1.37	327.00	2.57	740.00	--	--
	0.57	225.00	1.52	326.00	2.58	670.00	--	--
	0.58	232.00	1.37	320.00	2.67	683.00	--	--
AVG.	0.56	227.67	1.42	324.33	2.61	697.67	--	--
SD.	0.02	3.79	0.09	3.79	0.05	37.23	--	--
Bamboo	0.44	194.00	0.59	235.00	0.66	329.00	--	--
	0.45	202.00	0.62	244.00	0.64	328.00	--	--
	0.46	210.00	0.65	254.00	0.63	325.00	--	--
AVG.	0.45	202.00	0.62	244.33	0.64	327.33	--	--
SD.	0.01	8.00	0.03	9.50	0.01	2.08	--	--
S–Lyocell	0.52	205.00	--	--	--	--	--	--
	0.53	207.00	--	--	--	--	--	--
	0.53	208.00	--	--	--	--	--	--
AVG.	0.53	206.67	--	--	--	--	--	--
SD.	0.00	1.53	--	--	--	--	--	--

**Table 4:** Air permeability in cm^3^/cm^2^/s.

Fiber and Structure	SJ		Terry		Rib		Mesh	
	Avg.	SD.	Avg.	SD.	Avg.	SD.	Avg.	SD.
Polyester	190.2	9.6	114.8	2.9	229.8	7.1	151.5	2.9
Cotton	95.7	7.2	15.6	0.8	109.6	8.6	622.8	20.6
Elastane	527.5	29.7	101.8	6.3	117.2	13.5	-------	------
Lyocell	217.7	2.1	67.8	3.7	87.4	12.4	471.3	16.7
M–Rayon	106.4	6.7	126.0	4.1	121.5	9.2	102.1	2.1
Wool	85.5	6.7	223.5	7.3	137.5	8.7		
Bamboo	113.3	4.1	47.0	6.9	87.4	12.4		
S–Lyocell	95.4	2.5						

**Table 5:** Moisture management test.

		Wetting Time Top (s)	Wetting Time Bottom (s)	Top Absorption Rate (%/s)	Bottom Absorption Rate (%/s)	Top Max Wetted Radius (mm)	Bottom Max Wetted Radius (mm)	Top Spreading Speed (mm/s)	Bottom Spreading Speed (mm/s)	Accumulative One-Way Transport Index	OMMC	Grade (OMMC)
Polyester	SJ	3.84	4.31	62.59	71.01	21.67	25.00	4.01	3.58	23.57	0.47	3.00
	T	120.00	4.99	0.00	68.89	0.00	5.00	0.00	1.16	1154.73	0.69	4.00
	R	120.00	3.71	0.00	69.14	0.00	10.00	0.00	2.58	1170.39	0.80	4.00
	M	2.81	3.00	53.73	56.36	21.67	25.00	4.55	4.72	116.39	0.56	3.00
Cotton	SJ	9.55	7.71	62.25	100.01	20.00	20.00	2.32	2.95	154.21	0.63	4.00
	T	5.12	10.42	55.97	33.60	16.67	10.00	2.39	0.88	−429.26	0.07	1.00
	R	82.47	23.77	60.08	105.80	1.67	10.00	0.22	1.87	783.70	0.59	3.00
	M	81.47	4.71	8.56	65.73	1.67	10.00	0.36	3.16	1007.67	0.83	5.00
Elastane	SJ	4.43	4.46	70.53	62.74	25.00	25.00	4.71	4.60	−46.34	0.38	2.00
	T	4.40	4.74	63.99	58.92	15.00	15.00	2.17	2.07	−95.14	0.23	2.00
	R	15.76	7.39	57.70	122.27	13.33	13.33	0.80	1.27	264.06	0.52	3.00
	M	120.00	3.59	0.00	57.08	0.00	8.33	0.00	2.84	953.61	0.78	4.00
Lyocell	SJ	3.15	3.15	62.58	71.45	23.33	21.67	4.31	4.17	−20.87	0.45	3.00
	T	65.86	4.09	14.11	44.68	3.33	5.00	0.04	1.22	780.69	0.62	4.00
	R	13.60	10.11	227.97	42.66	5.00	5.00	0.43	0.57	484.13	0.50	3.00
	M	115.57	3.53	1.01	54.03	1.67	5.00	0.02	1.54	942.35	0.67	4.00
M–Rayon	SJ	82.59	74.20	38.82	6.53	1.67	5.00	0.21	0.20	−48.04	0.19	1.00
	T	120.00	3.40	0.00	55.14	0.00	5.00	0.00	1.64	1007.73	0.68	4.00
	R	4.62	4.81	66.67	58.17	15.00	15.00	2.31	2.21	−101.65	0.24	2.00
	M	1.53	2.12	62.44	60.00	25.00	25.00	11.02	10.12	-58.18	0.40	3.00
Wool	SJ	24.12	27.86	29.20	35.87	8.33	8.33	0.66	0.63	−12.34	0.13	1.00
	T	13.73	81.34	485.60	285.91	5.00	8.33	0.36	0.71	−746.04	0.25	2.00
	R	8.14	120.00	338.61	0.00	5.00	0.00	0.60	0.00	−1062.00	0.00	0.00
Bamboo	SJ	53.14	7.86	315.61	64.44	3.33	10.00	0.17	2.46	334.01	0.47	3.00
	T	28.11	7.21	167.78	68.53	8.33	11.67	0.28	2.66	639.58	0.80	4.00
	R	11.20	10.17	70.87	97.00	13.33	13.33	1.36	1.82	95.02	0.44	3.00
S–Lyocell	SJ	4.15	4.18	68.38	65.91	20.00	20.00	2.96	3.62	−48.44	0.38	2.00

Note: fabrication knit structures are as follows: SJ = single jersey; T = terry; R = rib; and M = mesh. Grade ratings are as follows: 1 = poor; 2 = fair; 3 = good; 4 = very good; 5 = excellent.

**Table 6:** Thermal and water vapor resistance.

Fiber	Structure	Avg RCF	SD	Avg REF	SD
		K.m^2^/W		Pa.m^2^/W	
Polyester	SJ	0.0088	0.0009	0.7233	0.2069
	Terry	0.0219	0.0009	1.5143	0.5269
	Rib	0.0237	0.0024	0.8693	0.3424
	Mesh	0.0127	0.0009	0.9343	0.1130
Cotton	SJ	0.0181	0.0014	2.3213	0.3531
	Terry	0.0358	0.0062	4.8803	0.1024
	Rib	0.0265	0.0019	0.6863	2.8847
	Mesh	0.0170	0.0002	2.3777	0.1640
Elastane	SJ	0.0105	0.0016	2.7417	0.2203
	Terry	0.0221	0.0019	3.0837	0.3352
	Rib	0.0173	0.0011	2.4887	0.0885
	Mesh	0.0043	0.0015	1.4797	0.9487
Lyocell	SJ	0.0155	0.0033	2.0457	0.4609
	Terry	0.0302	0.0033	4.0137	0.5299
	Rib	0.0233	0.0023	0.4843	1.4749
	Mesh	0.0113	0.0033	1.1720	1.8867
M–Rayon	SJ	0.0094	0.0023	2.9187	0.1955
	Terry	0.0241	0.0038	3.6897	0.2295
	Rib	0.0156	0.0036	2.2320	1.0648
	Mesh	0.0126	0.0023	1.1387	0.1465
Wool	SJ	0.0314	0.0015	3.8963	0.3365
	Terry	0.1276	0.0141	11.7137	0.6023
Bamboo	SJ	0.0131	0.0002	2.6603	0.5196
	Terry	0.0234	0.0007	3.9723	0.3398
	Rib	0.0159	0.0007	4.5673	0.2063
S–Lyocell	SJ	0.0177	0.0015	2.4250	0.4917

**Table 7:** Total thermal loss.

Fiber	Total Thermal Loss (W/m^2^)			
	SJ (1)	Terry (2)	Rib (3)	Mesh (4)
Polyester	1050.4	873.6	775.8	995.0
Cotton	785.3	558.0	1003.2	783.0
Elastane	770.1	703.2	770.5	942.5
Lyocell	824.0	617.7	1054.1	959.0
M–rayon	758.6	652.5	802.8	959.9
Wool	622.8	294.3	--	--
Bamboo	767.7	635.6	621.5	--
S–Lyocell	775.8	--	--	--

**Table 8:** Water vapor transmission in g/h·m^2^.

Structure	Polyester	Cotton	Elastane	Lyocell	M–Rayon	Wool	Bamboo	S–Lyocell
SJ	25.39	28.8	32.52	28.56	31.52	35.02	27.33	29.35
Terry	21.98	31.98	27.63	26.78	21.59	26.09	20.8	--
Rib	32.53	30.89	22.28	25.93	20.74	29.11	26.39	--
Mesh	26	29.26	30.98	25.39	26.39	--	--	--

Note: the water vapor permeability test was carried out at 25 °C, 50% RH, and in 800 mL water.

**Table 9:** Textile performance rankings considering PPE socks.

	Textile Rankings		
Performance Tests	1st	2nd	3rd
Air Permeability	Polyester SJ	Polyester Mesh	Wool Rib
Thermal Resistance	Elastane Mesh	Polyester SJ	Modal–Rayon SJ
Water Vapor Resistance	Lyocell Rib	Cotton Rib	Polyester Rib
Thermal Loss	Lyocell Rib	Polyester SJ	Polyester Mesh
Moisture Management	Polyester Rib	Cotton Mesh	Bamboo Terry
Water Vapor Transmission	Wool SJ	Polyester Rib	Elastane SJ
Antimicrobial	* Bamboo Rib	*--------*	*--------*

Note: * based on Arafa Badr’s (2018) [17] study.

## Data Availability

The original contributions presented in this study are included in the article. Further inquiries can be directed to the corresponding author.
